# Follicular Helper T Cells Remodel the Immune Microenvironment of Pancreatic Cancer via Secreting CXCL13 and IL-21

**DOI:** 10.3390/cancers13153678

**Published:** 2021-07-22

**Authors:** Xuan Lin, Longyun Ye, Xu Wang, Zhenyu Liao, Jia Dong, Ying Yang, Rulin Zhang, Hao Li, Pengcheng Li, Lei Ding, Tianjiao Li, Wuhu Zhang, Shuaishuai Xu, Xuan Han, Huaxiang Xu, Wenquan Wang, Heli Gao, Xianjun Yu, Liang Liu

**Affiliations:** 1 Department of Pancreatic Surgery, Shanghai Cancer Center, Fudan University, Shanghai 200032, China; linxuan@fudanpci.org (X.L.); yelongyun@fudanpci.org (L.Y.); wangxu2013@fudan.edu.cn (X.W.); liaozhenyu@fudanpci.org (Z.L.); dongjia@fudanpci.org (J.D.); yangying@fudanpci.org (Y.Y.); lihao@fudanpci.org (H.L.); lipengcheng@fudanpci.org (P.L.); dinglei@fudanpci.org (L.D.); litianjiao@fudanpci.org (T.L.); zhangwuhu@fudanpci.org (W.Z.); xushuaishuai@fudanpci.org (S.X.); hanxuan@fudanpci.org (X.H.); xuhuaxiang@fudanpci.org (H.X.); wangwenquan@fudanpci.org (W.W.); gaoheli@fudanpci.org (H.G.); 2 Department of Oncology, Shanghai Medical College, Fudan University, Shanghai 200032, China; 3 Shanghai Pancreatic Cancer Institute, Shanghai 200032, China; 4 Pancreatic Cancer Institute, Fudan University, Shanghai 200032, China; 5 Department of Laboratory Medicine, Shanghai General Hospital, Shanghai 200080, China; rulin_zhang@163.com

**Keywords:** follicular helper T cells, immune microenvironment, immunosuppression, neoadjuvant chemotherapy, pancreatic ductal adenocarcinoma

## Abstract

**Simple Summary:**

The immunosuppressive microenvironment is closely related to the poor prognosis of patients with PDAC. Tfh cells play an anti-tumor function in various malignant solid tumors; however, the role of Tfh cells in PDAC has not been determined. In this study, we aimed to explore the function of Tfh cells in PDAC, and revealed a novel immunosuppressive mechanism mediated by Tfh cells. Tfh cells promoted the formation of an immunoactive tumor microenvironment by secreting CXCL13 and IL-21, and the high infiltration of Tfh cells correlated with better patient prognosis. However, the anti-tumor function of Tfh cells was inhibited by the PD-L1/PD-1 signaling pathway. Neoadjuvant chemotherapy could further reverse the function of Tfh cells. Our results provided new strategies to remodel the immunoactive tumor microenvironment of PDAC.

**Abstract:**

Immunosuppression is an important factor for the poor prognosis of pancreatic ductal adenocarcinoma (PDAC). Follicular helper T cells (Tfh cells) play an anti-tumor role in various malignant solid tumors and predict better patient prognosis. In the present study, we aimed to determine the immunosuppressive mechanism associated with Tfh cells and explore a new strategy to improve the tumor microenvironment of PDAC. Flow cytometry was used to detect the infiltration and proportion of Tfh cells in tumor tissues and peripheral blood from patients with PDAC. The spatial correlations of Tfh cells with related immune cells were evaluated using immunofluorescence. The function of Tfh cells was examined using in vitro and in vivo model systems. The high infiltration of Tfh cells predicted better prognosis in patients with PDAC. Tfh cells recruited CD8^+^ T cells and B cells by secreting C-X-C motif chemokine ligand 13 (CXCL13), and promoted the maturation of B cells into antibody-producing plasma cells by secreting interleukin 21 (IL-21), thereby promoting the formation of an immunoactive tumor microenvironment. The function of Tfh cells was inhibited by the programmed cell death 1 ligand 1 (PD-L1)/programmed cell death 1 (PD-1) signaling pathway in PDAC, which could be reversed using neoadjuvant chemotherapy. Treatment with recombinant CXCL13, IL-21 and Tfh cells alleviated tumor growth and enhanced the infiltration of CD8^+^ T cells and B cells, as well as B cell maturation in a PDAC mouse model. Our results revealed the important role of Tfh cells in mediating anti-tumor cellular immunity and humoral immunity in PDAC via secreting CXCL13 and IL-21 and determined a novel mechanism of immunosuppression in PDAC.

## 1. Introduction

Pancreatic ductal adenocarcinoma (PDAC) is a threat to human life, being a highly invasive and metastatic malignancy [1]. PDAC is generally resistant to chemotherapy and immunotherapy, with a five-year overall survival rate of less than 10% [1,2]. PDAC is a typical “cold” tumor, characterized by an immunosuppressive microenvironment [2,3]. Previous studies have revealed that the absence and low activation of CD8^+^ T cells induced by the matrix composition, especially myeloid cell infiltration, contribute to the immunosuppressive microenvironment [4,5,6,7], indicating the significant roles of T cells in the PDAC immune microenvironment. However, the other cellular components in the microenvironment have barely been investigated. In particular, the anti-tumor role of B cells in establishing the immune response in PDAC has come to light recent years [8,9,10,11]; however, the precise regulatory mechanisms remain unexplored. In the present study, we aimed to investigate a novel follicular helper T cells (Tfh cells)-mediated mechanism of immunosuppression in PDAC.

Tfh cells are a subgroup of CD4^+^ T cells discovered in 2000, which are required helpers of B cell maturation and antibody production, playing a protective or pathogenic role in anti-infective immune responses or autoimmune diseases [12]. In addition, Tfh cells are a component of tumor-associated tertiary lymphoid structures (TLSs) [13] in the tumor microenvironment. In recent years, the infiltration of Tfh cells was discovered to correlate positively with better prognosis in patients with malignant tumors, which mainly depended on the recruitment of C-X-C motif chemokine ligand 13 (CXCL13) to CD8^+^ T cells and B cells [14,15,16,17,18]. In parallel, Tfh cells secrete the cytokine interleukin 21 (IL-21), which promotes the differentiation and maturation of B cells into plasma cells that produce tumor antigen-specific antibodies and thus, enhance the long-term anti-tumor immune response [15,19,20]. Therefore, we hypothesized that Tfh cells directly regulate CD8^+^ T cells and B cells to remodel the immune microenvironment in PDAC.

In the present study, we investigated the infiltration of Tfh cells in PDAC and uncovered their anti-tumor role using in vitro and in vivo model systems. The high infiltration of Tfh cells correlated positively with better prognosis in patients with PDAC. Tfh cells increased the infiltration of CD8^+^ T cells and B cells by secreting CXCL13, and promoted the maturation of B cells into plasma cells that produced tumor antigen-specific antibodies by secreting IL-21, thereby enhancing anti-tumor cellular immunity and the long-term humoral immunity response. Notably, the anti-tumor effect of Tfh cells was impaired by programmed cell death 1 ligand 1 (PD-L1)/programmed cell death 1 (PD-1) signaling, which could be reversed using neoadjuvant chemotherapy. Lastly, ectopic Tfh cells induced an immunoactive microstructure in mouse models of PDAC. Taken together, our results revealed an important role of Tfh cells in anti-tumor immunity and a novel Tfh-mediated mechanism of immunosuppression, providing a new strategy to transform PDAC from an immunosuppressive “cold” tumor to an immunologically active “hot” tumor.

## 2. Materials and Methods

### 2.1. Human Specimens

Tumor tissue specimens, peripheral blood samples, and spleens were acquired from Fudan University Shanghai Cancer Center. Intra-tumor tissues and peripheral blood samples were obtained from patients with PDAC during curative intent surgery. Spleens were acquired from patients with pancreatic tail cancer during surgery. All specimens were confirmed by postoperative pathology. The disease stage was defined according to the 8th edition American Joint Committee on Cancer (AJCC) stage system, and the multiple samples of different tumor stages used in the study were acquired from different patients. Detailed clinical data on the patient cohort are shown in Table S1.

Fragments (1–2 mm^3^) of fresh tumor tissue specimens were digested in Roswell Park Memorial Institute (RPMI) 1640 medium with Liberase TL (0.1 mg/mL, Roche Diagnostics, Indianapolis, IN, USA) and DNase I (10 U/mL, Roche Diagnostics) for 30 min. Single cells were filtered through 70 μm cell strainers (BD Biosciences, San Jose, CA, USA) and re-suspended in Percoll (40%, GE Healthcare, Chicago, IL, USA) for gradient centrifugation. Peripheral blood samples were diluted in phosphate-buffered saline (PBS) and added to Ficoll (GE Healthcare) for gradient centrifugation to obtain peripheral blood mononuclear cells (PBMCs). Spleens were ground on a 70 μm cell strainer, filtered, and re-suspended in red blood cell lysate (BD Biosciences) to remove red blood cells.

### 2.2. Mice

Male C57BL/6 mice (5 weeks old) purchased from Shanghai Jihui Co. (Shanghai, China) were bred under the specific pathogen-free (SPF) conditions. The armpit of each mouse was injected subcutaneously with 1 × 10^7^ PANC-2 cells in 200 μL of PBS (pH 7.4). Three days later, the mice in the control group were injected with PBS, and the mice in the three experimental groups were injected with recombinant mouse (rm) CXCL13 (5 μg, Peprotech, Rocky Hill, NJ, USA), rmIL-21 (5 μg, Peprotech) and 1 × 10^5^ Tfh cells, separately, via the tail vein every 3 days and euthanized 2 weeks later. The mice weight and tumor measurements were conducted every two days and the tumor volume (mm^3^) was estimated as (length × width^2^)/2. Tumors were harvested and either digested into a single-cell suspension for flow cytometry analysis or fixed in formaldehyde for immunohistochemistry (IHC) of mouse CD8 (1:300; Abcam, Cambridge, MA, USA) and mouse CD19 (1:300; Invitrogen, Waltham, MA, USA). The animal studies were conducted under protocols approved by the Ethics Committee of Fudan University Shanghai Cancer Center (ethic code: FUSCC-IACUC-S20210159, approved at 23 February 2021).

### 2.3. Flow Cytometry and Antibodies

Dead cells were excluded using Fixable Viability Dye eFluor780 (eBioscience, San Diego, CA, USA). Nonspecific staining was blocked using Fc Receptor Blocker (93, BD Biosciences). Intracytoplasmic staining and intranuclear transcriptional factor staining was performed according to the manufacturer’s instructions (BD Biosciences). For intracellular CXCL13 and IL-21 staining, cells were stimulated with Leukocyte Activation Cocktail (BD Biosciences) for 6 h. The following antibodies were used for the human specimens: anti-CD45 (HI30), anti-CD45RO (UCHL1), anti-CD4 (GK1.5), anti-CD25 (BC96), anti-CXCR5 (SPRCC5), anti-PD1 (29F-1A12), anti-ICOS (C398.4A), anti-Foxp3 (259D/C7), anti-Bcl6 (K112-91), anti-CXCL13 (53610), anti-IL-21 (3A3-N2.1), anti-CD8 (SK1), anti-CD19 (H), anti-CD20 (2H7), anti-B220 (RA3-6B2), anti-CD27 (0323), anti-CD38 (HIT2), anti-IgD (11-26c.2a), anti-CD138 (281-2), anti-IgG (QA19A42), and anti-IgM (MHM88). The following antibodies were used for the mouse specimens: anti-CD45 (30-F11), anti-CD4 (RM4-5), anti-CXCR5 (L138D7), anti-PD1 (29F.1A12), anti-ICOS (C398.4A), anti-CD8 (53-5.8), anti-B220 (RA3-6B2), and anti-CD138 (281-2). All antibodies were purchased from BD Bioscience and Biolegend (San Diego, CA, USA).

## 3. Immunofluorescent Staining and Immunohistochemistry

Paraffin-embedded formalin-fixed PDAC tumor tissue sections were cut into 5 μm sections and processed for immunohistochemistry and immunofluorescent staining. For immunohistochemistry, human tumor sections were incubated with anti-CD4 (1:200, ab133616, Abcam), anti-CXCL13 (1:200, AF801, R&D), anti-IL-21(1:200, ab5978, Abcam), anti-CD8 (1:300, ab237709, Abcam), and anti-CD20 (1:300, ab78237, Abcam) antibodies; mouse tumor sections were incubated with anti-CD8 (1:500, ab209775, Abcam), anti-CD20 (1:500, ab122788, Abcam), and anti-CD138 (1:200, ab181789, Abcam) antibodies. For immunofluorescence, human tumor sections were incubated with anti-CD4 (1:200, ab133616, Abcam), anti-CXCR5 (1:200, ab46218, Abcam), anti-PD-1 (1:200, ab53587, Abcam), anti-CXCL13 (1:50, AF801, R&D), anti-IL-21(1:100, ab5978, Abcam), anti-CD8(1:500, ab237709, Abcam), and anti-CD20 (1:200, ab78237, Abcam) antibodies. Sections were visualized under a fluorescence microscope (NIKON ECLIPSE C1, Tokyo, Japan), and images were acquired using a digital camera (NIKON DS-U3). The tumor-infiltrating lymphocytes (TILs) in the immunohistochemistry and immunofluorescence analyses were defined as the lymphocytes inside the tumor.

## 4. Cell Purification and Differentiation In Vitro

CD4^+^ T cells, CD8^+^ T cells, and B cells were purified using isolation kits according to the manufacturer’s protocols (STEMCELL Technologies, Vancouver, BC, Canada). For B cell maturation detection, Tfh cells (CD4^+^ CD25^−^ CXCR5^+^ PD-1^+^) and naive B cells (CD19^+^ CD27^−^ IgD^+^) were sorted using a FACSAria II flow cytometer (BD) and co-cultured at a ratio of 1:1 in serum-free T cell medium containing 1 μg/mL endotoxin-reduced staphylococcal enterotoxin B (SEB; Sigma, St. Louis, MO, USA). Cells were collected for cytokine detection on day 12. For the in vitro differentiation experiments, Tfh cells were stimulated using anti-CD3/CD28/CD2 antibodies (STEMCELL Technologies). For the chemotaxis assay, CD8^+^ T cells (5 × 10^5^) and B cells (5 × 10^5^) were seeded in a Transwell chamber (3 μm, Costar, Corning Inc., Corning, NY, USA), and Tfh cells (1 × 10^6^) were seeded at the bottom of the chamber, with or without recombinant human (rh) CXCL13 (Abcam) or anti-CXCL13 neutralizing antibody (R&D System, Minneapolis, MN, USA).

## 5. Statistical Analysis

Statistical analyses were performed using Prism software (version 6, GraphPad Software, Inc., La Jolla, CA, USA). Significant differences data between the two groups were compared using a Student’s *t*-test. Overall survival (OS) and progression-free survival (PFS) of patients with PDAC were analyzed using the Kaplan–Meier method log-rank test. Data are presented as means ± SDs. *p*-values < 0.05 were considered to be statistically significant, and asterisks indicated the significance level of the *p*-value (* *p* < 0.05, ** *p* < 0.01, *** *p* < 0.001 and **** *p* < 0.0001) in the figures.

## 6. Results

### 6.1. Tumor-Infiltrating Tfh Cells Are Beneficial for the Prognosis of Patients with PDAC

**Figure 1:**
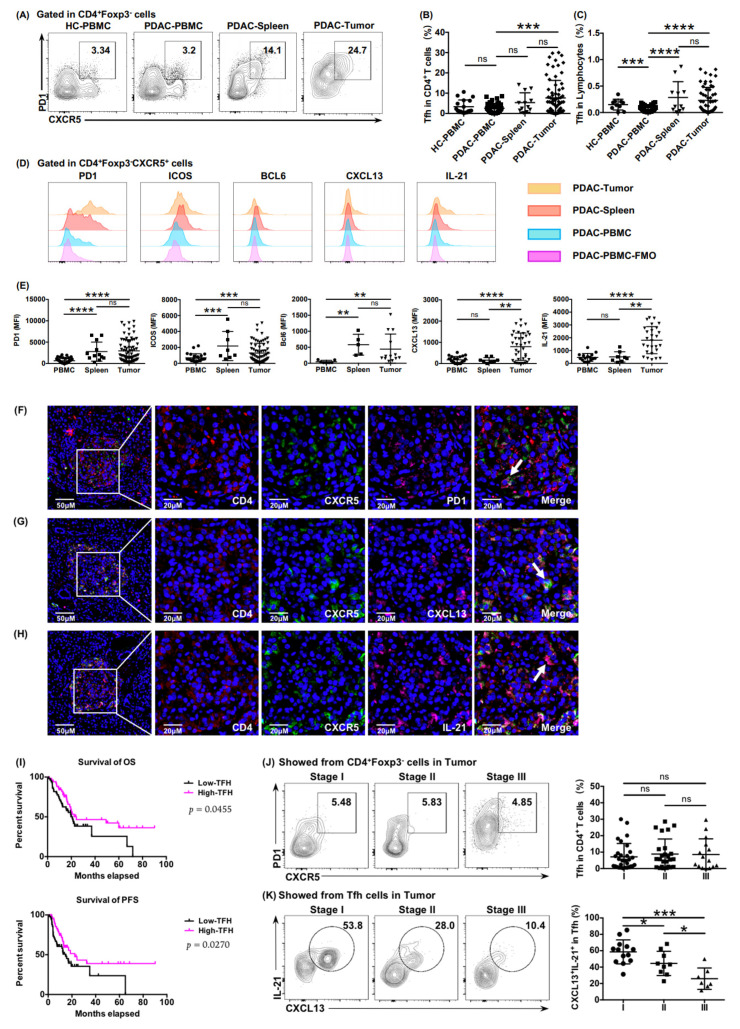
Tumor-infiltrating Tfh cells correlated positively with the prognosis of patients with PDAC. (**A**) Plots showing representative CXCR5 and PD1 surface staining of Tfh cells from peripheral blood (healthy donors and patients with PDAC), tumor tissue (patients with PDAC), and spleen (patients with PDAC). (**B**) Proportion of Tfh cells in CD4^+^ T cells from peripheral blood (healthy donors, *n* = 18; patients with PDAC, *n* = 45), tumor tissue (patients with PDAC, *n* = 12), and spleen (benign pancreatic cystic neoplasms, *n* = 69). (**C**) Proportion of Tfh cells in lymphocytes from peripheral blood (healthy donors, *n* = 10; patients with PDAC, *n* = 48), tumor tissue (patients with PDAC, *n* = 11), and spleen (benign pancreatic cystic neoplasms, *n* = 57). (**D**) Representative histograms and (**E**) a statistical graph of the mean fluorescence intensity (MFI) of characteristic markers of Tfh cells from the peripheral blood, tumor tissues, and spleens of patients with PDAC. FMO: fluorescence minus one. (**F**–**H**) Representative immunofluorescence staining of total Tfh cells (CD4^+^ CXCR5^+^ PD1^+^) and functional Tfh cells (CD4^+^ CXCR5^+^ CXCL13^+^ and CD4^+^ CXCR5^+^ IL-21^+^) in tumor tissues of PDAC. The samples were stained for CD4 (red), CXCR5 (green), PD1 (pink), CXCL13 (pink), IL-21 (pink), and DAPI (blue). Scale bars: 50 or 20 μm. (**I**) Kaplan–Meier survival curves for overall survival (OS) (left) and progression-free survival (PFS) (right) in patients with PDAC from the training cohort according to the functional Tfh cells (CD4^+^ CXCR5^+^ CXCL13^+^) density (*n* = 127, log-rank test and *p* values are shown). (**J**) Representative flow cytometry data and the proportion of Tfh cells (CD4^+^ CXCR5^+^ PD1^+^) in CD4^+^ T cells in tumor tissue from patients with PDAC at different stages (*n_I_* = 30, *n_II_* = 25, *n_III_* = 15). (**K**) Representative flow cytometry data and the proportion of functional Tfh cells (CXCL13^+^ IL-21^+^) in Tfh cells in tumor tissue from patients with PDAC at different stages (*n_I_* = 30, *n_II_* = 25, *n_III_* = 15). Asterisks indicated the significance level of the *p*-value (ns: no significant difference, * *p* < 0.05, ** *p* < 0.01, *** *p* < 0.001 and **** *p* < 0.0001).

To investigate the presence of Tfh cells in PDAC, lymphocytes from peripheral blood, spleen, and tumor tissue of patients with PDAC were separated and examined using flow cytometry. Immunosuppressive follicular regulatory T cells (Tfr cells) expressing CD25 and Foxp3 have co-expressed molecules with Tfh cells; therefore, we gated Tfh cells (CD4^+^ CXCR5^+^ PD1^+^) from CD4^+^Foxp3^−^ cells (Figure S1A, Figure 1A). The individual difference in Tfh infiltration in tumor tissue was significantly stronger than that in peripheral blood and the spleen (Figure 1B,C). We then tested other characteristic molecules related to Tfh cells such as ICOS, Bcl6, CXCL13, and IL-21 (Figure 1D). Surface markers (PD1 and ICOS) had similar expression panels in tumors and spleens (Figure 1E), and significantly different expression levels of functional effectors (CXCL13 and IL-21) reflected the diverse status of tumor-infiltrating Tfh cells exposed to tumor antigens (Figure 1E).

The immunofluorescence results showed that the infiltration of Tfh cells in the tumor tissues of PDAC (Figure 1F–H), and in patients with high infiltration of functional Tfh cells (CD4^+^ CXCR5^+^ CXCL13^+^), resulted in longer OS and PFS (Figure 1I), indicating a positive correlation between functional infiltrating Tfh cells and patient survival. We further examined the relationship between functional infiltrating Tfh cells and the progression of PDAC. The expression of functional factors CXCL13 and IL-21 decreased gradually in higher tumor stages (Figure 1K), although the proportion of tumor-infiltrating Tfh cells among CD4^+^ T cells showed no significant reduction (Figure 1J), which suggested that the function of Tfh cells was impaired in the tumor microenvironment of PDAC.

### 6.2. Tumor-Infiltrating Tfh Cells Were Related to CD8^+^ T Cell and B Cell Infiltration

**Figure 2:**
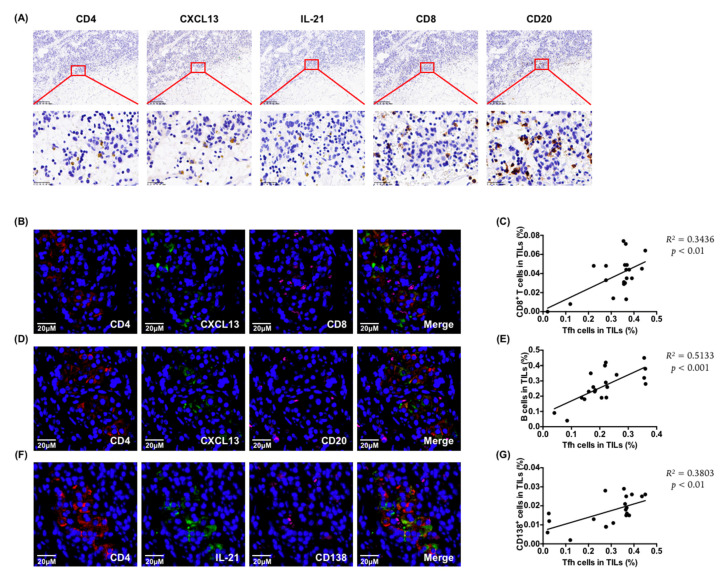
Tumor-infiltrating Tfh cells were related to CD8^+^ T cells and B cells infiltration. (**A**) Representative images of immunohistochemistry for CD4^+^, CXCL13^+^, IL-21^+^, CD8^+^, and CD20^+^ cells of continuous slices of tumor tissue from patients with PDAC. Scale bars: 200 μm or 25 μm. (**B**) Representative images of the immunofluorescence of the co-localization of functional tumor-infiltrating Tfh cells (CD4^+^ CXCL13^+^) with CD8^+^ T cells. Scale bars: 20 μm. (**C**) Correlation between functional tumor-infiltrating Tfh cells (CD4^+^ CXCL13^+^) with CD8^+^ T cells (*n* = 20). (**D**) Representative images of immunofluorescence of the co-localization of functional tumor-infiltrating Tfh cells (CD4^+^ CXCL13^+^) with CD20^+^ B cells. Scale bars: 20 μm. (**E**) Correlation between functional tumor-infiltrating Tfh cells (CD4^+^ CXCL13^+^) with CD20^+^ B cells (*n* = 20). (**F**) Representative images of the immunofluorescence of the co-localization of functional tumor-infiltrating Tfh cells (CD4^+^ IL-21^+^) with CD138^+^ plasma cells. Scale bars: 20 μm. (**G**) Correlation between functional tumor-infiltrating Tfh cells (CD4^+^ IL-21^+^) and CD138^+^ plasma cells (*n* = 20).

To explore the role of tumor-infiltrating Tfh cells, we examined the relationship between Tfh cells and other immune cells in PDAC tumor tissues. The immunohistochemistry results showed that the distribution of functional effectors CXCL13 and IL-21 of Tfh cells co-localized with CD8^+^ T cells and B cells (Figure 2A), and the immunofluorescence analysis showed that the CD8^+^ T cells (Figure 2B) and CD20^+^ B cells (Figure 2D) were adjacent to functional Tfh cells (CD4^+^ CXCL13^+^). Moreover, there was a positive correlation between the infiltration of Tfh cells and CD8^+^ T cells (Figure 2C). This was consistent with the analysis results from the public database [21], which showed that the infiltration of Tfh cells correlated strongly with the ratio of total CD8^+^ T cells and cytotoxic cells in TILs (Figure S2A). In addition, the infiltration of functional Tfh cells correlated positively with the distribution of CD20^+^ B cells (Figure 2D,E). Moreover, functional tumor-infiltrating Tfh cells (CD4^+^ IL-21^+^) correlated positively with the infiltration of CD138^+^ plasma cells (Figure 2F,G). Taken together, these observations support a potential role for infiltrating Tfh cells in promoting anti-tumor immunity in PDAC.

### 6.3. Tfh Cells Mediated Anti-Tumor Cellular Immunity and Humoral Immunity, Which Was Impaired in PDAC

**Figure 3:**
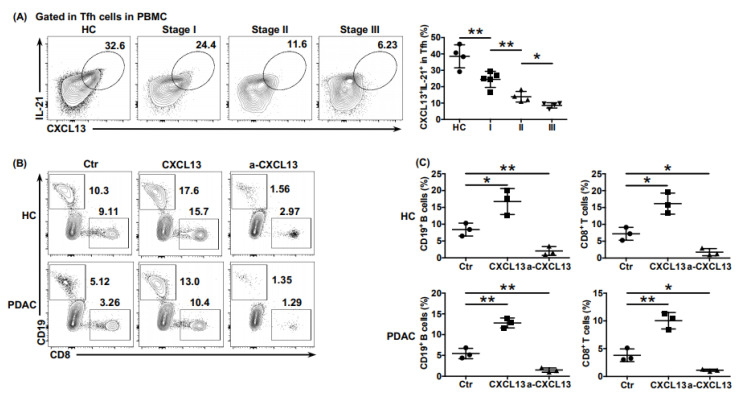

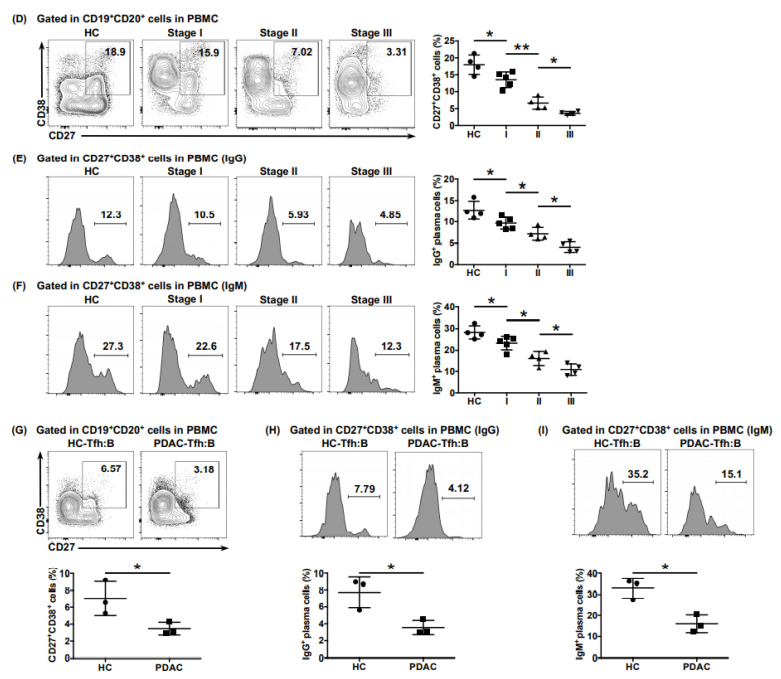
The anti-tumor function of Tfh cells was impaired in PDAC. (**A**) Representative flow cytometry data and the proportion of functional cTfh cells (CXCL13^+^ IL-21^+^) from the peripheral blood of healthy donors (*n* = 4) and patients with PDAC of different stages (*n_I_* = 5, *n_II_* = 4, *n_III_* = 4). (**B**) Representative flow cytometry data and (**C**) the proportion of CD8^+^ T cells and B cells recruited to the bottom of the Transwell chamber by cTfh cells treated with or without rhCXCL13 or anti-CXCL13 neutralizing antibodies. (**D**) Representative flow cytometry data and the proportion of plasma cells (CD27^+^ CD38^+^) in B cells (CD19^+^ CD20^+^) from the peripheral blood of healthy donors (*n* = 4) and patients with PDAC at different stages (*n_I_* = 5, *n_II_* = 4, *n_III_* = 4). (**E**) Representative flow cytometry data and the proportion of IgG-secreting plasma cells from cells in (**D**). (**F**) Representative flow cytometry data and the proportion of IgM-secreting plasma cells from cells in (**D**). (**G**) Representative flow cytometry data and the proportion of plasma cells (CD27^+^ CD38^+^) in B cells (CD19^+^ CD20^+^) co-cultured with cTfh cells sorted from the peripheral blood of healthy donors (*n* = 3) and patients with PDAC (*n* = 3). (**H**) Representative flow cytometry data and the proportion of IgG-secreting plasma cells from cells in (**G**). (**I**) Representative flow cytometry data and the proportion of IgM-secreting plasma cells from cells in (**G**). Asterisks indicated the significance level of the *p*-value (* *p* < 0.05 and ** *p* < 0.01).

To further explore the potential anti-tumor role of Tfh cells in PDAC, we detected the function of circulating Tfh cells (cTfh cells) in the peripheral blood of healthy donors and patients with PDAC. Compared with that in healthy donors, the ability of cTfh cells to express effector factors CXCL13 and IL-21 in patients with PDAC was significantly impaired and was further impaired in higher tumor stages (Figure 3A). To verify the effect of CXCL13 secreted by Tfh cells on CD8^+^ T cells and B cells, cTfh cells (CD45RO^−^ CD4^+^ CD25^−^ CXCR5^+^ PD-1^+^) from healthy donors and patients with PDAC were sorted using flow cytometry. After amplification in vitro, Tfh cells with or without rhCXCL13 or anti-CXCL13 neutralizing antibody were added to the bottom of Transwell chambers of control group and experimental groups, and CD8^+^ T cells and B cells isolated from the peripheral blood of healthy donors were seeded in the top of the Transwell chambers of three groups at a ratio of 1:1. After 24 h, the percentage of CD8^+^ T cells and B cells in the bottom of the wells were detected. Whether in PDAC patients or healthy donors, the recruitment of CD8^+^ T cells and B cells significantly increased when treated with rhCXCL13 and was almost blocked when treated with anti-CXCL13 antibodies (Figure 3B,C), proving the crucial role of CXCL13 in recruiting CD8^+^ T cells and B cells. Notably, chemotaxis was significantly decreased in cells from PDAC patients compared with those from healthy donors, whether in vehicle conditions or when treated with rhCXCL13 (Figure S3C), and rhCXCL13 treatment roughly raised the recruitment level of CD8^+^ T cells and B cells of PDAC to the level of the untreated healthy control (Figure 3B,C). This indicated an impaired function of Tfh cells in PDAC, explaining why there were not many CD8^+^ T cells adjacent to functional Tfh cells (CD4^+^ CXCL13^+^) in Figure 2.

Additionally, we found that the proportions of total plasma cells (CD19^+^ CD20^+^ CD27^+^ CD38^+^) (Figure 3D) and of those secreting IgG (Figure 3E) and IgM (Figure 3F) in PDAC were significantly lower than those in the healthy donors, which gradually decreased in higher tumor stages. We further detected the auxiliary role of Tfh cells on B cell maturation by co-culturing cTfh cells from healthy donors or patients with PDAC with heterologous naive B cells (CD19^+^ CD27^−^ IgD^+^) at a ratio of 1:1, respectively. After 12 days, the proportions of total plasma cells (Figure 3G) and of those secreting IgG (Figure 3H) and IgM (Figure 3I) in B cells co-cultured with cTfh cells from patients with PDAC were significantly lower than those from the healthy donors, indicating the impaired auxiliary effect of Tfh cells on B cell maturation.

Taken together, these results indicated that Tfh cells induced the chemotaxis of CD8^+^ T cells and B cells by secreting the chemokine CXCL13 and promoted the maturation of B cells into plasma cells, which produced tumor antigen-specific antibodies, thereby enhancing PDAC impaired anti-tumor cellular immunity and anti-tumor humoral immunity.

### 6.4. The Impaired Function of Tfh Cells Was Mediated by PD-L1/PD-1 Signaling in PDAC

**Figure 4:**
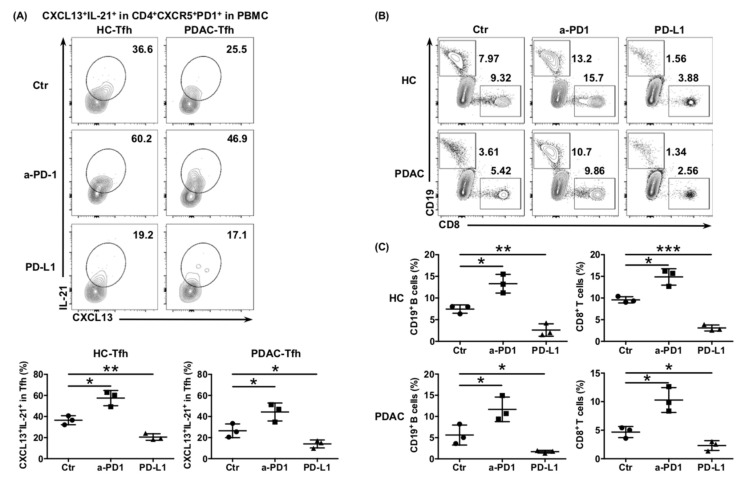

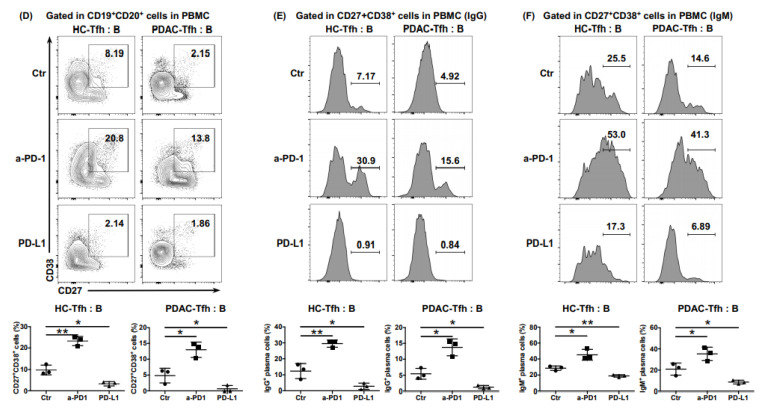
PD-L1/PD-1 signaling inhibits Tfh function and mediates immune escape of PDAC. (**A**) Representative flow cytometry data and the proportion of functional cTfh cells (CD4^+^ CXCR5^+^ PD1^+^ CXCL13^+^ IL-21^+^) cultured in in vitro with or without anti-PD1 or rhPD-L1 from the peripheral blood of healthy donors (*n* = 4) and patients with PDAC (*n* = 4). (**B**) Representative flow cytometry data and (**C**) the proportion of CD8^+^ T cells and B cells recruited to the bottom of the Transwell chamber by cTfh cells, treated with or without anti-PD1 or rhPD-L1 from the peripheral blood of healthy donors (*n* = 4) and patients with PDAC (*n* = 4). (**D**) Representative flow cytometry data and the proportion of plasma cells (CD27^+^ CD38^+^) in B cells (CD19^+^ CD20^+^) co-cultured with cTfh cells, treated with or without anti-PD1 or rhPD-L1 from the peripheral blood of healthy donors (*n* = 3) and patients with PDAC (*n* = 3). (**E**) Representative flow cytometry data and the proportion of IgG-secreting plasma cells from the cells in (**D**). (**F**) Representative flow cytometry data and the proportion of IgM-secreting plasma cells from the cells in (**D**). Asterisks indicated the significance level of the *p*-value (* *p* < 0.05, ** *p* < 0.01 and *** *p* < 0.001).

Tfh cells show persistently high expression of PD-1 on their surface, which is one of the characteristics that differentiates Tfh cells from other subtypes of CD4^+^ T cells. Considering that elevated PD-L1 expression correlates with poor prognosis in PDAC [22], we hypothesized that the function of Tfh cells would be inhibited by the abundant PD-L1/-PD1 signaling in the PDAC tumor environment. The ability of cTfh cells cultured in vitro to express CXCL13 and IL-21 was significantly enhanced when treated with anti-PD-1 antibodies and significantly inhibited by treatment with rhPD-L1, whether from healthy donors or patients with PDAC (Figure 4A). A similar trend was also observed in the ability of cTfh cells to recruit CD8^+^ T cells and B cells (Figure 4B,C), as well as to help B cells mature into plasma cells and produce antibodies in the Tfh:B cell co-culture system (Figure 4D–F). These results indicated that the function of Tfh cells was inhibited by PD-L1/PD-1 signaling, thereby mediating immunosuppression in PDAC.

### 6.5. Ectopic Tfh Cells Shaped the Immunoactive Tumor Microenvironment in Mouse Models of PDAC

**Figure 5:**
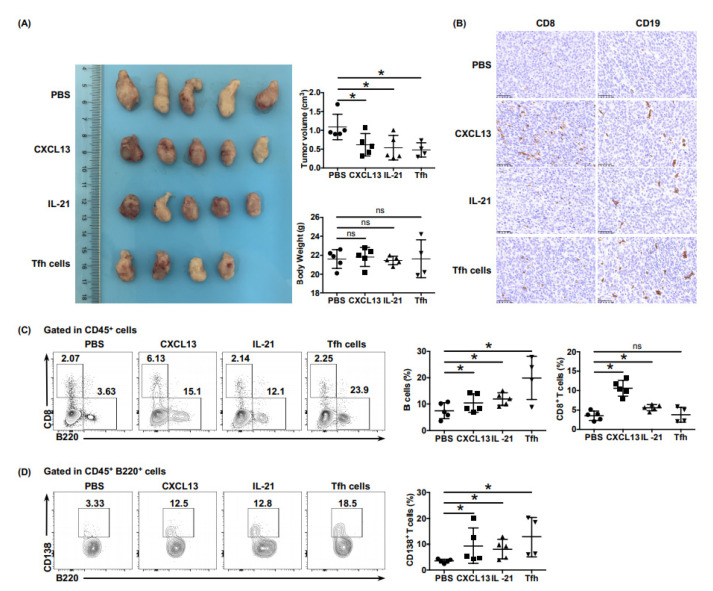
Tfh cells shape the immunoactive tumor microenvironment in a PDAC mouse model. (**A**) Resected tumors and tumor volumes in the subcutaneous PDAC mouse model treated with injection of PBS (*n* = 5), rmCXCL13 (*n* = 5), rmIL-21 (*n* = 5), or Tfh cells (*n* = 4), separately. (**B**) Representative images of immunohistochemistry of CD8^+^ T cells and CD20^+^ B cells of tumor tissues of tumor-bearing mice. Scale bars: 50 μm (**C**) Representative flow cytometry data and the proportions of CD8^+^ T cells and B cells in the lymphocytes of tumor tissues of tumor-bearing mice. (**D**) Representative flow cytometry data and the proportions of CD138^+^ plasma cells in the lymphocytes of tumor tissues of tumor-bearing mice. Asterisks indicated the significance level of the *p*-value (ns: no significant difference, * *p* < 0.05).

To accurately simulate the role of Tfh cells in the tumor microenvironment, we established a subcutaneous mouse model of PDAC. Every three days, PBS, rmCXCL13, rmIL-21, or Tfh cells isolated from the spleen of pancreatic cancer mice by flow cytometry were intravenously injected into tumor-bearing mice in the control group and three experimental groups, respectively. Compared with that of the PBS group, the tumor growth of the other groups was significantly inhibited, with no significant difference in weight when euthanized (Figure 5A). The results of immunohistochemistry and flow cytometry showed significantly more infiltration of CD8^+^ T cells and B cells in the rmCXCL13 group than in the PBS group (Figure 5B,C). Meanwhile, the proportion of CD138^+^ plasma cells in the rmIL-21 group and Tfh cells group was significantly higher than in the PBS group (Figure 5D). The above results revealed the role of CXCL13 in CD8^+^ T cell and B cell recruitment, and IL-21 in B cell maturation, which further proved that Tfh cells help to shape the immunoactive tumor microenvironment, thereby exerting anti-tumor effects in PDAC.

### 6.6. Neoadjuvant Chemotherapy Reversed the Dysfunction of Tfh Cells

**Figure 6:**
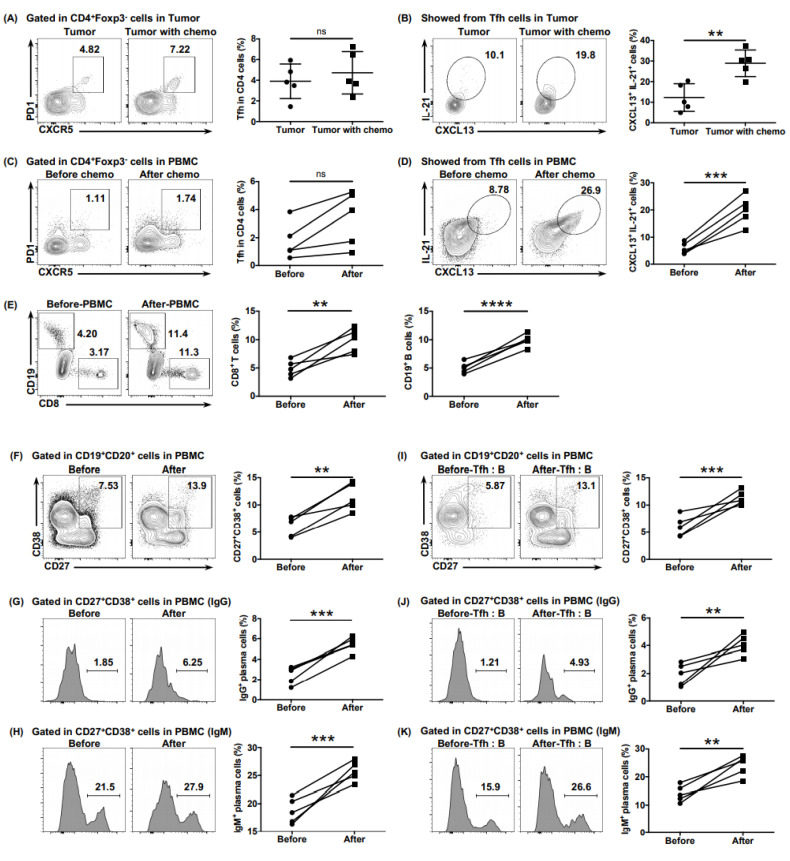
Neoadjuvant chemotherapy rescues the impaired function of Tfh cells. (**A**) Representative flow cytometry data and the proportion of total tumor-infiltrating Tfh cells (CD4^+^ CXCR5^+^ PD1^+^) from the tumor tissues of patients with PDAC with (*n* = 5) or without (*n* = 5) neoadjuvant chemotherapy. (**B**) Representative flow cytometry data and the proportion of functional tumor-infiltrating Tfh cells (CXCL13^+^ IL-21^+^) from the tumor tissues of patients with PDAC with (*n* = 5) or without (*n* = 5) neoadjuvant chemotherapy. (**C**) Representative flow cytometry data and (**F**) the proportion of total cTfh cells (CD4^+^ CXCR5^+^ PD1^+^) from the peripheral blood of patients with PDAC (*n* = 5) before or after receiving neoadjuvant chemotherapy. (**D**) Representative flow cytometry data and (**H**) the proportion of functional cTfh cells (CXCL13^+^ IL-21^+^) from the peripheral blood of patients with PDAC (*n* = 5) before or after receiving neoadjuvant chemotherapy. (**E**) Representative flow cytometry data and the proportion of CD8^+^ T cells and B cells recruited to the bottom of the Transwell chamber by the cTfh cells of patients with PDAC (*n* = 5) before or after receiving neoadjuvant chemotherapy. (**F**) Representative flow cytometry data and the proportion of plasma cells (CD27^+^ CD38^+^) in B cells (CD19^+^ CD20^+^) from the peripheral blood of patients with PDAC (*n* = 5) before or after receiving neoadjuvant chemotherapy. (**G**) Representative flow cytometry data and the proportion of IgG-secreting plasma cells from the cells in (**F**). (**H**) Representative flow cytometry data and the proportion of IgM-secreting plasma cells from the cells in (**F**). (**I**) Representative flow cytometry data and the proportion of plasma cells (CD27^+^ CD38^+^) in B cells (CD19^+^ CD20^+^) co-cultured with cTfh cells sorted from the peripheral blood of patients with PDAC (*n* = 5) before or after receiving neoadjuvant chemotherapy. (**J**) Representative flow cytometry data and the proportion of IgG-secreting plasma cells from the cells in (**I**). (**K**) Representative flow cytometry data and the proportion of IgM-secreting plasma cells from the cells in (**I**). Asterisks indicated the significance level of the *p*-value (ns: no significant difference, ** *p* < 0.01, *** *p* < 0.001 and **** *p* < 0.0001).

Considering the poor responsiveness of PDAC to immunotherapy, we speculated whether neoadjuvant chemotherapy would ameliorate the anti-tumor function of Tfh cells, thereby increasing the immune activity of the PDAC microenvironment. Samples from patients who had received four cycles of neoadjuvant AG (nab-paclitaxel + gemcitabine) before surgery were obtained. The levels of CXCL13 and IL-21 expressed by tumor-infiltrating Tfh cells in patients who received neoadjuvant chemotherapy was higher than those in patients who had not (Figure 6B), although the proportion of Tfh cells showed no obvious difference (Figure 6A). The ability of cTfh cells to express CXCL13 and IL-21 was enhanced significantly after receiving neoadjuvant chemotherapy (Figure 6D), with the proportion of cTfh cells showing no obvious increase (Figure 6C). A chemotaxis assay demonstrated an enhanced ability of cTfh cells after chemotherapy to recruit CD8^+^ T cells and B cells (Figure 6E). Consistently, the proportions of total plasma cells and of those secreting IgG and IgM increased significantly after chemotherapy, whether in B cells from peripheral blood (Figure 6F–H) or in those co-cultured with the Tfh cells of patients (Figure 6I–K), showing a reversal of the function of Tfh cells after neoadjuvant chemotherapy.

## 7. Discussion

For a long time, CD8^+^ T cells and NK cells have been considered the main force in killing tumor cells [23,24]; however, research has shown that, without sufficient assistance from CD4^+^ T cells, CD8^+^ T cells cannot reach their full potential, emphasizing the irreplaceable role of CD4^+^ T cells in anti-tumor immunity [25,26]. As a subgroup of CD4^+^ T cells, Tfh cells can assist in B cell differentiation and maturation and promote the production of antigen-specific antibodies [12]. In the past decade, Tfh cells have been extensively studied in the anti-infective immune response and in autoimmune diseases [14]. However, the most exciting discovery is the anti-tumor effects of Tfh cells in malignant tumors, including breast cancer, colorectal cancer, hepatocellular carcinoma, and non-small cell lung cancer [14,15,16,17,18]. In the present study, we revealed that Tfh cells indeed existed in the tumor tissues of PDAC, in which they remodel the anti-tumor microenvironment and correlate with tumor stages and better patient prognosis.

Recently, the role of CXCL13 in shaping the immunoactive tumor microenvironment has been confirmed [27,28,29,30]. In the study of Bai et al. [31], CD8^+^CXCR5^+^ T cells were identified as a potent subset of CD8^+^ T cells in PDAC, and over half of tumor-infiltrating CD8^+^ T cells were CD8^+^CXCR5^+^ T cells. Considering that CXCL13 is the only ligand for CXCR5, Tfh cells are likely to be the main contributor to the increased infiltration of CD8^+^ T cells, which could provide a prerequisite for the reactivation of exhausted CD8^+^ T cells under humorally active conditions. Mouse Tfh cells do not secrete CXCL13 [14]; therefore, we could not simulate the anti-tumor process of human CXCL13 secreting Tfh cells in a mouse model. Instead, we explored the method of intravenous injection of CXCL13 into one of the experimental groups of mice to intervene in tumor growth. Indeed, rmCXCL13 could recruit CD8^+^ T cells and B cells and thus, control tumor growth, which further proved that Tfh cells can remodel the anti-tumor microenvironment function through CXCL13 in pancreatic cancer. Additionally, we simulated the anti-tumor effects of IL-21 secreted by Tfh cells to promote B cell maturation and antibody production in the other two experimental groups of mice. However, repeated injections of CXCL13 or IL-21 might produce systemic spikes of cytokine activity, which might not necessarily mimic the biological reality of cytokine production by Tfh cells; therefore, it is necessary to verify the in vivo function of Tfh cells in the specific environments of PDAC using an optimized mouse model.

TLSs have been a hotspot in the field of tumor immunology in recent years [13]. TLSs are important sites of T-B cell interactions, and their presence correlates with good prognosis across multiple tumor types [32,33,34]. In PDAC, the existence of tumor-associated TLSs usually indicates a longer anti-tumor immune response and better prognosis [8,11]. TLS formation in PDAC is critical for a successful immune response; however, the mechanism of TLS formation is unknown. The infiltration of CD8^+^ T cells and B cells in PDAC is closely related to TLSs [11,35]. Notably, our immunofluorescence results showed that Tfh cells tended to infiltrate in the microstructures aggregated with immune cells (Figure 1F–H, left). Although the infiltration of B cells in these microstructures was not sufficient to identify them as the typical TLSs (data not shown), our observations confirmed the recruitment function of Tfh cells toward CD8^+^ T cells and B cells, hinting at a role for tumor-infiltrating Tfh cells in early-stage TLS formation, which is worthy of further in-depth exploration.

Persistently high expression of PD-1 is one of the characteristics of Tfh cells that distinguish them from other CD4^+^ T cell subgroups. In the germinal center, the expression of a normal level of PD-L1 can effectively prevent the overactivation of Tfh cells. Autoimmunity or poor-quality antibody responses could be caused by PD-1 deficiency [36,37]. However, in the immunosuppressive microenvironment, with an elevated level of PD-L1 in PDAC, the persistently high expression of PD-1 might lead to the function of Tfh cells being inhibited, representing a tool by which tumor cells achieve immune escape. In the present study, we found that Tfh cells promoted anti-tumor cellular immunity by increasing the infiltration of CD8^+^ T cells, and mediated anti-tumor humoral immunity by increasing the infiltration of B cells and promoting the maturation of B cells into plasma cells. Therefore, we hypothesized that the release of Tfh-mediated immunosuppression by immune checkpoint inhibitors could benefit patients with PDAC in terms of both anti-tumor cellular immunity and humoral immunity. Owing to the limited size of tumor samples, we could only verify the hypothesis using circulating Tfh cells from the peripheral blood of patients with PDAC. However, the mechanism by which the function of tumor-infiltrating Tfh cells is inhibited in PDAC deserves further research.

At present, the curative effect of immunotherapy for pancreatic cancer in the clinic is unsatisfactory. In view of the complex tumor microenvironment of pancreatic cancer, compared with immune checkpoint inhibitors or other immunotherapy methods, endogenous expansion and activation of tumor-infiltrating anti-tumor immune effector cells might represent a more effective strategy to establish a long-term and stable anti-tumor immune response. Further research on the anti-tumor effect of Tfh cells will help to deepen our understanding of the immunosuppressive mechanism in the tumor microenvironment of pancreatic cancer. Reversing the function of Tfh cells might promote the establishment of an immunoactive tumor microenvironment, as well as inducing a long-term anti-tumor immune response, which would be helpful to transform “cold tumors” into “hot tumors”.

## 8. Conclusions

Taken together, Tfh cells played anti-tumor role via CXCL13-dependent recruitment of CD8^+^ T cells and B cells and IL-21-dependent B cell maturation. However, the anti-tumor function of Tfh cells was impaired by PD-L1 /PD-1 signaling in PDAC, which could be reversed using neoadjuvant chemotherapy. Our results uncovered a novel mechanism of immunosuppression mediated by Tfh cells, and provided new strategies for pancreatic cancer immunotherapy.

## Data Availability

The data presented in this study are available on request from the corresponding author. The data are not publicly available due to privacy.

## Supplementary Materials

The following are available online at https://www.mdpi.com/article/10.3390/cancers13153678/s1, Figure S1. (A)The strategy of gating Tfh cells, taking the peripheral blood sample as an example. Figure S2. (A) Analysis result of correlation between functional Tfh cells with CD8^+^ T cells from the public database (http://bioinfo.life.hust.edu.cn/web/ImmuCellAI/). Figure S3. (A) Representative flow cytometry data and (B) the proportion of Tfh cells (CD4^+^ CXCR5^+^ PD1^+^) in CD4^+^ T cells from the peripheral blood of healthy donors (*n* = 14) and patients with PDAC at different stages (*n_I_* = 11, *n_II_* = 13, *n_III_* = 13). (C) Comparison of the proportion of CD8^+^ T cells and B cells recruited to the bottom of the Transwell chamber by cTfh cells between HC and PDAC for conditions treated with or without rhCXCL13 or anti-CXCL13 neutralizing antibodies. Table S1. Demographics and clinical characteristics of the cohort of patients with PDAC from whom were obtained fresh intra-tumor tissues and peripheral blood samples from (*n* = 62) in the study.
