# Supplementary material for: Follicular Helper T Cells Remodel the Immune Microenvironment of Pancreatic Cancer via Secreting CXCL13 and IL-21
Source: Cancers (Basel). 2021 Jul 22;13(15):3678. doi: 10.3390/cancers13153678 (PMC8345153; doi:10.3390/cancers13153678)
Supplement: Supplementary file 1 [file cancers-13-03678-s001.zip › cancers-1266885-supplementary.pdf]

cancers

# Follicular Helper T Cells Remodel the Immune Microenvironment of Pancreatic Cancer via Secreting CXCL13 and IL-21

**Xuan Lin et al.**

**Table S1.** Demographics and clinical characteristics of the cohort of patients with PDAC from whom were obtained fresh intra-tumor tissues and peripheral blood samples from (*n* = 62) in the study.

| Characteristics | Number | Percentage (%) |
|---|---|---|
| Sex | | |
| Male | 32 | 51.6 |
| Female | 30 | 48.4 |
| Age (years) (median) | 52 | / |
| Tumor size (mm) | | |
| Mean (SD) | 30.5 | 2.0 |
| Median (range) | 29.1 | 6.0–95.0 |
| Location | | |
| Head | 36 | 58.1 |
| Others | 26 | 41.9 |
| Lymph node positive | | |
| Yes | 27 | 43.5 |
| No | 35 | 56.5 |
| AJCC$^{8th}$ TNM stage | | |
| I | 23 | 37.1 |
| II | 20 | 32.2 |
| III | 19 | 30.6 |
| With chemotherapy * | | |
| Yes | 5 | 8.1 |
| No | 57 | 91.9 |

AJCC8$^{th}$, 8th edition American Joint Committee on Cancer; TNM, tumor-node-metastasis. * Except for the five patients in Figure 6 who had received neoadjuvant chemotherapy before surgery, none of the patients received chemotherapy before surgery.

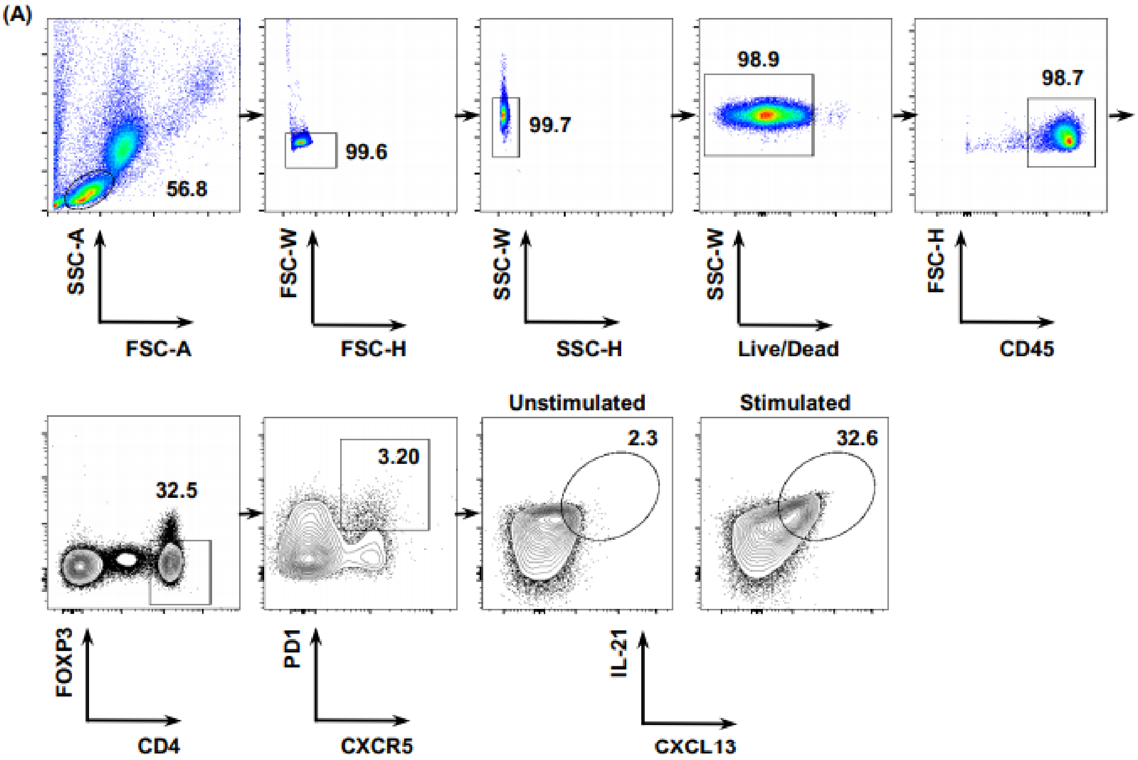


**Figure S1. (A)**The strategy of gating Tfh cells, taking the peripheral blood sample as an example.

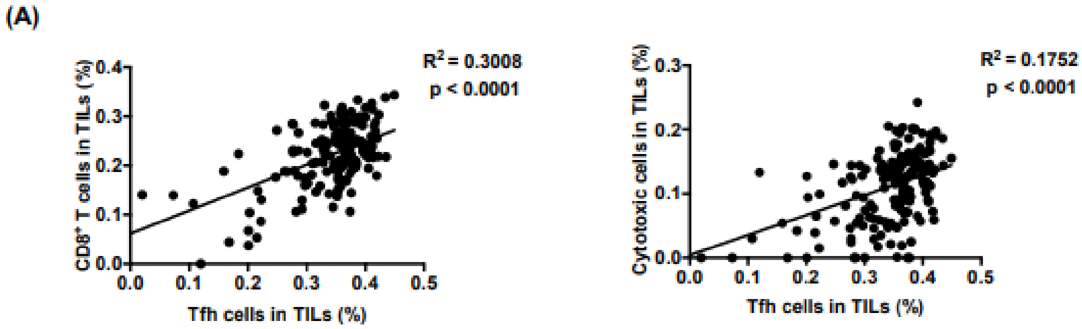


**Figure S2.** (A) Analysis result of correlation between functional Tfh cells with CD8⁺ T cells from the public database (http://bioinfo.life.hust.edu.cn/web/ImmuCellAI/).

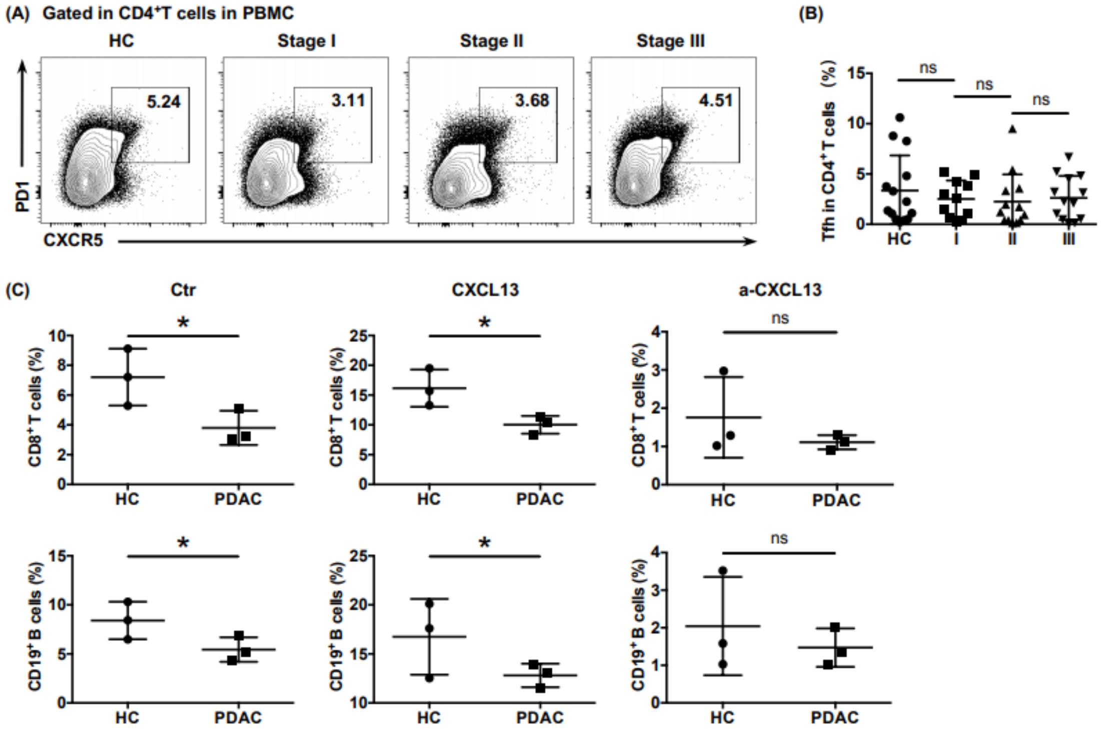


**Figure S3.** (A) Representative flow cytometry data and (B) the proportion of Tfh cells ($CD4^+$ $CXCR5^+$ $PD1^+$) in $CD4^+$ T cells from the peripheral blood of healthy donors ($n$ = 14) and patients with PDAC at different stages ($n_I$ = 11, $n_{II}$ = 13, $n_{III}$ = 13). (C) Comparison of the proportion of $CD8^+$ T cells and B cells recruited to the bottom of the Transwell chamber by cTfh cells between HC and PDAC for conditions treated with or without rhCXCL13 or anti-CXCL13 neutralizing antibodies.
